# Canada’s northern food subsidy *Nutrition North Canada*: a comprehensive program evaluation

**DOI:** 10.1080/22423982.2017.1279451

**Published:** 2017-02-02

**Authors:** Tracey Galloway

**Affiliations:** ^a^ Department of Anthropology, University of Toronto Mississauga, Mississauga, Canada

**Keywords:** Food security, north, Arctic, Indigenous, community, food cost, hunger, policy

## Abstract

**Background**: Nutrition North Canada (NNC) is a retail subsidy program implemented in 2012 and designed to reduce the cost of nutritious food for residents living in Canada’s remote, northern communities. The present study evaluates the extent to which NNC provides access to perishable, nutritious food for residents of remote northern communities.

**Design**: Program documents, including fiscal and food cost reports for the period 2011–2015, retailer compliance reports, audits of the program, and the program’s performance measurement strategy are examined for evidence that the subsidy is meeting its objectives in a manner both comprehensive and equitable across regions and communities.

**Results**: NNC lacks price caps or other means of ensuring food is affordable and equitably priced in communities. Gaps in food cost reporting constrain the program’s accountability. From 2011–15, no adjustments were made to community eligibility, subsidy rates, or the list of eligible foods in response to information provided by community members, critics, the Auditor General of Canada, and the program’s own Advisory Board. Measures to increase program accountability, such as increasing subsidy information on point-of-sale receipts, make NNC more visible but do nothing to address underlying accountability issues

**Conclusions**: The current structure and regulatory framework of NNC are insufficient to ensure the program meets its goal. Both the volume and cost of nutritious food delivered to communities is highly variable and dependent on factors such as retailers’ pricing practices, over which the program has no control. It may be necessary to consider alternative forms of policy in order to produce sustainable improvements to food security in remote, northern communities.

Nutrition North Canada (NNC) is a retail subsidy designed to provide residents in 128 isolated northern communities with reliable, affordable access to nutritious, perishable food. In communities reliant on air freight shipments for perishable food supply, the subsidy is paid directly to retailers who sell eligible foods in local stores. The program is administered in Ottawa under the jurisdiction of Indigenous and Northern Affairs Canada (INAC).^1^ ^1^INAC, the federal Ministry responsible for the administration of northern food subsidy policy, has undergone a series of name changes. Its legal title is set out under the Department of Indian Affairs and Northern Development Act [50], though from 2011–2015 it was known as Aboriginal Affairs and Northern Development Canada. In this paper, the acronym INAC will be used to indicate the activities and publications of this Ministry across these time periods. Implemented in 2011, NNC replaced the Food Mail program, a transportation subsidy on northern goods delivered through Canada Post Corporation.

In the year following the NNC implementation, the program’s Advisory Board raised numerous concerns brought forward by community members who suspected they were not receiving the full benefit of the subsidy [1]. In April 2013, the United Nations (UN) Special Rapporteur on the Right to Food, Olivier de Schutter, expressed similar concerns in a report to the UN General Assembly: “the federal Government has no way of verifying if the subsidy is being passed on, despite the obligation imposed on subsidy recipients to attest that they have complied with this requirement” [2].[p. 18] A review of the NNC program by the Auditor-General of Canada raised a number of concerns related to community eligibility and program management, and reiterated claims that the program lacked accountability:

Overall, we found that [INAC] has not verified whether the northern retailers pass on the full subsidy to consumers. The Department has not required the information it needs to verify this in the contribution agreements it has signed with northern retailers. It also has not required that compliance reviews of northern retailers include analysis of profit margins in order to verify that the full subsidy is being passed on [3].[p. 5]

Canada’s northern food subsidy operates within a context of severe food insecurity. Statistics Canada estimates the prevalence of food insecurity in Canada’s northern territories is 11.4% in Yukon, 13.7% in Northwest Territories, and 36.7% in Nunavut, compared to 8.3% in Canada as a whole (Figure 1) [4]. The 2012 Aboriginal People’s Survey found rates of food insecurity and hunger in northern Inuit communities ranged from 32% in Northwest Territories to 56% in Nunavut [5]. While a number of regional initiatives have been launched to combat the high rate of food insecurity, the federal subsidy remains the primary national policy response to this ongoing challenge.

**Figure 1.**
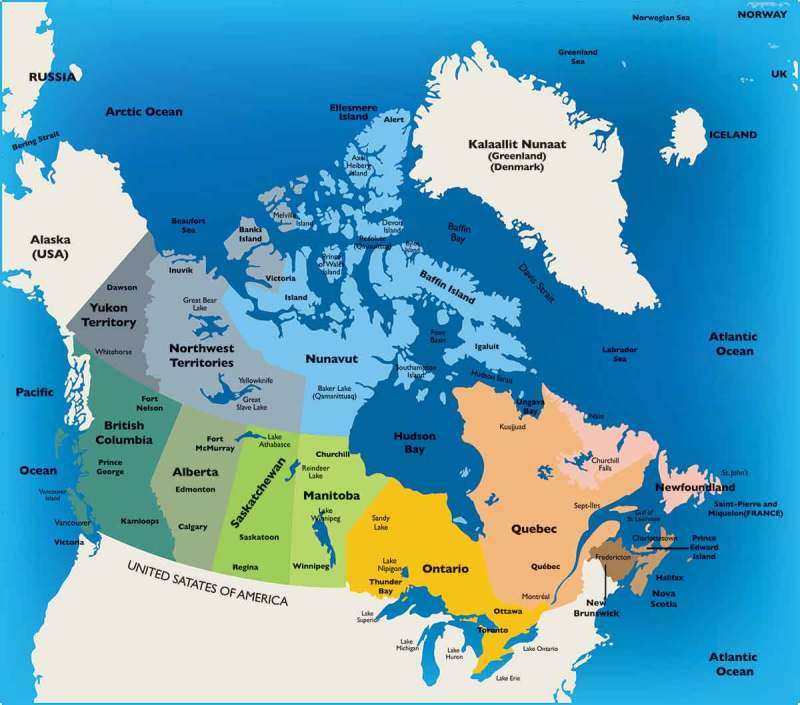
Map of Canada, provinces and territories. *Image credit: Esra Ogunday Bakir/shutterstock.com. Reproduced with permission.

In circumpolar terms, Canada’s food subsidy policy appears to be unique. For example, Alaska operates a food stamp program, with the state responsible for 50% of the cost of distributing federally-funded food stamp vouchers directly to residents [6]. Benefits are assessed on a sliding scale taking into account such factors as age, income, and community remoteness. Greenland employs a strict regulatory framework for pricing healthy food. Its *Kalaallit Niuerfiat* chain of suppliers includes the state-run *Pilersuisoq* stores which provide food at regulated prices in the country’s smaller towns and villages [7].

In May 2016, Canada’s Minister for Indigenous and Northern Affairs, the Honourable Carolyn Bennett, began a series of public engagement sessions designed to guide improvements to Canada’s food subsidy policy [8]. The present study is a response to that initiative, and is designed to provide a detailed, third-party review of the NNC program which can inform both the consultation process and the development of subsidy program frameworks in Canada and other jurisdictions that serve remote communities.

According to the National Collaborating Centre for Healthy Public Policy, there is opportunity for various public health actors to influence policy-making at four stages of the policy-making process: agenda setting, policy formation, implementation, and policy evaluation [9]. The present study seeks to bridge the third and fourth stages [9] by accomplishing the following:

- documenting the consequences of previously adopted policies
- producing analyses, applying technical skills and expert knowledge with an emphasis on the possibility of applying evidence gathered across different contexts
- revealing discrepancies between the policy’s expected and actual results [9]

The purpose of this paper, then, is to provide an independent and comprehensive evaluation of the NNC program. The objective is twofold: 1. To assess the extent to which NNC is achieving its stated intent of improving access to nutritious, perishable food in remote northern communities; 2. To illustrate how the subsidy’s structure, embedded in a market-oriented policy framework, constrains its ability to achieve its stated goal. The intent is that this information will inform and improve future iterations of food subsidy policy so that a more rigorous accountability mechanism and a more equitable food cost structure are achieved, ultimately contributing to sustainable improvements in food security for Canada’s northern residents.

## Methods

### Conceptual framework

The present study employs a modified conceptual framework adapted from Hardee et al [10] with an emphasis on how policy and programs act to improve health outcomes. The focus is on identifying particular aspects of programs that influence program performance and equity of outcomes.

### Materials

The analysis relies primarily on data gathered by the NNC program itself, and incorporates a limited amount of additional data available from external sources. The following data sources are currently available on the NNC program website (http://www.nutritionnorthcanada.gc.ca/):

- lists of eligible communities and subsidy levels
- quarterly and annual fiscal reports for fiscal years (01 April to 31 March) 2011–12 through 2014–15; these include subsidy expenditures and kilogram weights by product category, province/territory, community, per capita, and retailer;
- quarterly reports on the cost of the Revised Northern Food Basket (RNFB) 2011–12 through 2014–15
- retailer compliance reports for fiscal years 2011–12 through 2013–14
- the NNC Performance Measurement Strategy

Additional data are supplied by external reports available on various government and non-governmental websites: internal and external evaluations conducted since 2011, including *From Food Mail to Nutrition North Canada: A Report of the Standing Committee on Aboriginal Affairs and Northern Development* [11]; a 2013 internal program audit [12]; the 2014 Fall Report of the Auditor-General of Canada [3]; and evaluations conducted by the NNC Advisory Board in 2012 [1] and the Nunavut Food Security Coalition in 2015 [13]. Supplementary information is provided from annual price reports compiled by the Nunavut Bureau of Statistics 2013–2015 [14]. Finally, this study draws on media reports published 2011–2016 to highlight relevant issues affecting community members and other stakeholders.

### Analytic approach

Information is compiled and presented for the program as a whole and for participating provinces/territories, and communities in order to judge program performance and equity. Descriptive statistics are used to compare results among provinces/territories and communities. Results are compared with the program mandate and with performance outcomes articulated in the program’s Performance Measurement Strategy [15].

Once data are examined for evidence that NNC is producing its intended outcomes, a second level of analysis is employed to determine whether any failure to achieve intended outcomes arises from the process of program implementation or whether it reflects the overarching program structure or policy environment. In particular, this analysis examines NNC’s system of monitoring and accountability, and examines whether the evidence collected by program officials constitutes sufficient means of evaluating the efficacy of the program.

## Results

### Program overview

NNC is a retail subsidy program designed to subsidise the freight costs borne by retailers and other suppliers who ship perishable, healthy food to remote northern communities. In eligible communities, retailers who record retail sales of items from a Health Canada-approved list of healthy, perishable foods may receive subsidy payments to offset their freight costs, provided they enter into legally-binding contribution agreements with INAC. These contribution agreements stipulate that retailers submit monthly electronic statements to INAC. Retailers are also subject to routine compliance reports and program audits by INAC and the Auditor General of Canada. An Advisory Board provides ongoing consultation with northern stakeholders and communities and guidance to the Minister and program staff regarding the operation of the subsidy. The program was designed to operate within fixed budget of $60 million per year [16].

### Community eligibility

Eligibility for the program is based on the community’s use of the old Food Mail subsidy between April 2009 and March 2010: a community is deemed eligible for “full subsidy” if it received over 15,000 kg of perishable food over that period; it is eligible for “partial subsidy” if it received between 100 and 14,999 kg of perishable food over that period [17]. These criteria for eligibility were reported to a parliamentary standing committee in January 2011:

INAC…explained that the decision to limit the definition of “isolated northern communities” eligible under the NNC program was taken to reflect the fact that not all “communities that are not accessible year-round by road, rail or marine service” are isolated to the same degree, as some communities did not require a subsidy under the Food Mail program. As well, to ensure that NNC resources were focused on the most northerly and remote communities, the definition of “isolated northern community” was qualified using 2009–2010 shipment data [11].[p. vii]

According to INAC officials, annual review of community eligibility is a key feature of the program: “Community eligibility levels (full vs. nominal) will be re-evaluated annually by INAC based on analysis of food prices in the communities” [11].[p. 2] From 2011 to July 2016, the NNC website listed 103 eligible communities: 84 eligible for full subsidy and 19 eligible for partial subsidy [17]. With the sole addition of Pauingassi MB, which became eligible for full subsidy in August 2012, the list of eligible communities was not altered between April 2011 and July 2016.^2^ ^2^On 18 July 2016, the Government of Canada announced that the list of eligible communities would be expanded by an additional 37 communities [22]. The expanded eligibility list includes a combination of 25 newly-eligible communities (the majority of these are in MB and ON) and 12 communities whose eligibility status has been raised from partial- to full-subsidy eligibility. The changes will came into in October 2016.


It is, in fact, not communities themselves but rather companies operating grocery stores with retail sales in eligible communities that may enrol in the NNC subsidy program. Retailers wishing to collect subsidy payments must sign contribution agreements with INAC which require them to produce monthly air freight forecasts, monthly itemised air freight shipment invoices and matching waybills, and monthly food price reports on a pre-determined list of items [18]. They must also participate in nutrition education activities and promote program visibility on cash register receipts and through in-store signage and displays. Adherence to these requirements is monitored using two means: food cost reporting and retailer compliance reviews.

### Direct orders

Also eligible to receive subsidy are private individuals who purchase eligible items ordered through a list of approved suppliers. These direct or “personal” orders were a popular feature of the old Food Mail program [19] and were retained in the current subsidy program due to pressure from community members [11]. In its 2012 report on the subsidy’s first year of implementation, the NNC Advisory Board cited concerns from community members over limited access to the direct order option:

…the direct order option is not available to all Northerners since in many cases direct ordering requires a credit card and financial means to order larger quantities of eligible foods at one time [1].[p. 19]

The list of approved direct order suppliers includes Valu Lots, a Winnipeg retailer owned by North West Company [20]. Over the period 2011–15, customers making direct order purchases received between 2.4% and 3% of total subsidy funds annually [21].

### Subsidy rates applied under the NNC program

**Figure 2.**
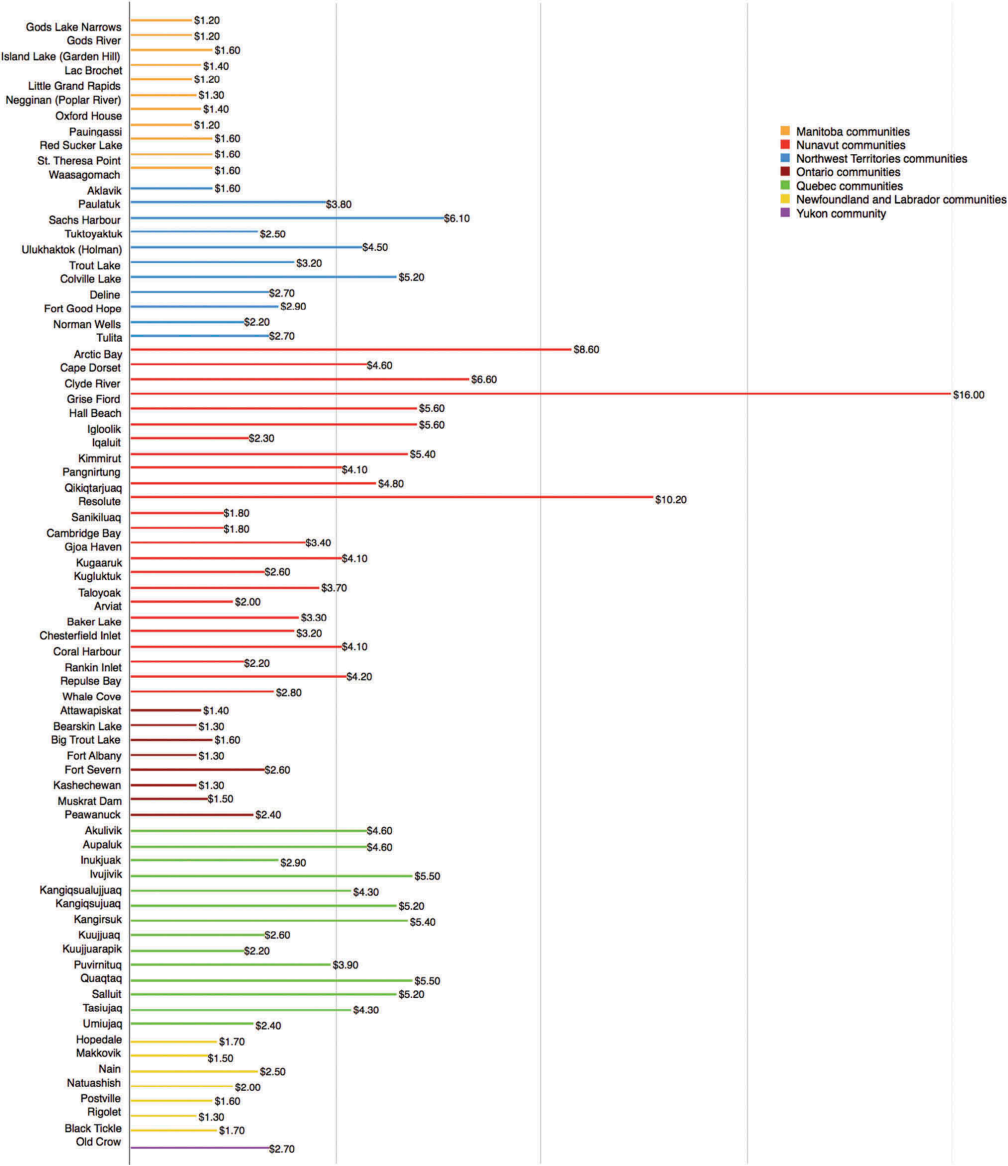
Level 1 subsidy rates for communities eligible for full subsidy* by province/territory [17]. *Data for Quebec North Shore communities are not included as they are served by NNC for short periods only when there is no marine service available.

Subsidy is allocated on a per kilogram basis to retailers who can verify sales of these items in eligible communities. Level 1 (higher) and level 2 (lower) subsidy rates are provided for all eligible communities, however subsidy rates are significantly lower in communities eligible for partial subsidies (Figure 2). The NNC website states: “Subsidy rates are reviewed periodically and may be adjusted during the year” [16]. The program’s Performance Measurement Strategy, published in 2014, indicates that mechanisms are in place to support subsidy level review: “…data can be used to support funding forecasts, program and policy reviews, and adjustments (including adjustments to the subsidy model, the list of eligible food and rates)” [15].[p. 14] The NNC Advisory Board Report of 2012 contains a similar statement: “Going forward, food prices will also be used to inform adjustments to subsidy rates, with the goal of achieving a measure of equitability across all eligible communities” [1].[p. 18]

Despite these statements, subsidy rates have not altered since they were introduced in 2011, nor is there publicly-available evidence of regular review of subsidy rates or response to changing conditions in freight or fuel costs. The Report of the Auditor General of Canada examined this issue in detail:

When the Program was introduced in 2011, subsidy rates for each eligible community were based on freight rates at that time. Under the contribution agreements, retailers are expected to use the most effective and cost-efficient supply chain arrangements and routes, in order to reduce the price of eligible items as much as possible and to provide the best quality for consumers. Some northern retailers publicly reported that they were able to lower their freight rates. The Department also told us that the subsidy rate is now higher than the freight rate in some communities [3].[p. 14]

The Auditor-General recommended program officials consider revisions to subsidy rates in an effort to contain program costs. In response INAC committed to “examine all options, including annual changes to the subsidy rates, with a view to avoiding unintended price shocks or product shortages” [3].[p. 14] After a gap of 20 months, on 18 July 2016, the Government of Canada announced that the list of eligible communities would be expanded, with the changes to take effect 1 October 2016 [22]. Subsidy rates for these newly-qualified communities have not yet been published.

### Subsidised foods

Subsidy payments are provided for a list of food items developed in conjunction with Health Canada and phased in over an 18-month period 2011–2012 (Table 1) [23]. Eligible foods are categorised according to *Canada’s Food Guide for First Nations, Inuit and Métis* [24] with additional categories for “other foods” and non-prescription drugs. Higher, level 1 subsidy items include a wide range of perishable food items including: fresh and frozen fruits and vegetables; bread and cereals; milk, cheese and yogurt; eggs; fresh and frozen meat, fish and poultry; and traditional or “country” food items when available. Lower, level 2 subsidy items include non-prescription drugs, frozen fruit juice, bacon, and a number of items necessary for cooking or baking: flour, cooking oil, margarine, butter, and shortening. Due to significant differences between the eligible food lists of Food Mail and NNC, a transitional eligible foods list was implemented in April 2011 [1]. The NNC list of eligible foods took effect in October 2012.

**Table 1.** List of subsidised foods* by subsidy level [23].

	Higher “Level 1” Subsidy	Lower “Level 2” Subsidy
Vegetables and Fruit	fresh and frozen vegetables and fruit dried vegetables and fruit (unseasoned or unsweetened) frozen unsweetened juice concentrate unsweetened juice in individual containers of 250 ml or less, except cans	unsweetened juice in containers larger than 250 ml, except cans
Grain products	bread and bread products without filling or coating ready-to-eat cereals cook-type cereals	flour crackers, dry crisp breads and Pilot biscuits arrowroot and social tea cookies fresh pasta, without sauce
Milk and Alternatives	milk (e.g. fresh, UHT, powdered, canned evaporated) buttermilk fortified soy beverages cheese and processed cheese slices cottage cheese yogurt and yogurt drinks	cream sour cream cream cheese processed cheese spread ice cream, ice milk, sherbet, sorbet and frozen yogurt
	fresh and frozen meat, poultry, fish and seafood eggs and egg substitutes unsweetened nuts and seeds peanut butter and other nut or seed based spreads “vegetarian” products (e.g. tofu, vegetable-based patties)	side bacon
Country or Traditional Foods	country or traditional foods when available through local stores or when purchased from processing plants that are registered with the program	
Other Foods	infant formula, infant cereals and other infant foods	margarine, butter, lard and shortening salad dressing, mayonnaise and dips fresh, frozen and refrigerated combination foods *except* items that are breaded, battered or in pastry, desserts, poutine, prepared sandwiches, hamburgers, hot dogs, prepared salads cooking oils
Non-food Items		non-prescription drugs

*An expanded list of eligible foods is available for the community of Old Crow YT

During a 2011 parliamentary review of the transition from Food Mail to NNC, committee members expressed the view that INAC should reconsider retaining subsidy for a range of food and non-food items deemed essential in northern communities: child care products (diapers, baby food etc.); traditional hunting and related food supplies (gasoline, ammunition, high-fat food consumed during harvesting activities); and various dried foods used as convenient and affordable complements to traditional cooking practices (dried soups, pasta, rice etc) [11]. In its 2012 report, the NNC Advisory Board cited the following concerns among community members about the NNC foods list:

- Dropping diapers from the eligibility list will have a major impact on young families.
- There were questions about removal of snowmobile parts from the list since this facilitates the harvest of country food.
- There was disappointment that detergent is not on the eligibility list.
- There was concern medical supplies and prescription drugs are no longer on the eligibility list.
- The eligibility list must have a few compromise items (e.g. less nutritious perishable items such as ice cream).
- Back bacon is eligible but not commonly used in northern communities. Side bacon is used to flavour stews and other foods, but not necessarily eaten on its own.
- The fact that larger cans of juice receive a lower level of subsidy while individual-sized juice boxes are eligible for the higher subsidy creates confusion and concern [1].[p. 10]

As a result of these concerns, the NNC eligible foods list was adjusted to accommodate non-prescription drugs, ice cream and side bacon (level 2 subsidy) [23]; the remaining concerns were not addressed. A 2015 report by the Nunavut Food Security Coalition criticised the subsidy for privileging fresh bread products at the expense of baking supplies:

In Nunavut communities, where many Inuit may prefer to make bannock or bread rather than purchase bread, flour receives a lower level of subsidy than prepared bread. In addition, other bannock ingredients such as oil and lard are subsidised at the lower level, and basic baking staples such as yeast, baking soda and baking powder are not subsidised at all [13].[p. 11]

The report called for INAC to revise the eligible foods list to better reflect the preferences and food habits of northerners. A March 2014 price survey conducted by the Nunavut Bureau of Statistics reported high prices and considerable variability in many of the items mentioned in critics’ concerns [14]. A bulk package of 60 disposable diapers (an unsubsidised item) cost $32.57, with prices ranging from $24.99 in Kugluktuk to $45.99 in Resolute. Shampoo (375 mL, unsubsidised item) cost $8.93 in Chesterfield Inlet and $13.99 in Igloolik. Cooking oil (a level 2 subsidy item) ranged in price from $9.60/litre in Kugluktuk to $14.99/litre in Hall Beach. The cost of a 10 kilogram bag of all-purpose flour (a level 2 subsidy item) ranged from $28.99 in Whale Cove to $53.62 in Clyde River.

The NNC program subsidises freight costs for traditional or “country” food. In order for these foods to be eligible for subsidy, they must either be:

- shipped by plane from registered Country food processors/distributors and processed in government regulated and/or approved-for-export commercial plants or
- shipped by plane from the South by a registered Northern retailer or Southern supplier and subsidised at the same level as other store-bought meat (level 1 subsidy) in the eligible food list [23]

The total subsidy funds allocated for country food under the NNC program is provided in annual fiscal reports [21]. Over the period 2011–2015, country food accounted for less than 0.1% of subsidy expenditures annually (Table 2).

**Table 2.** Country food as a proportion of annual NNC subsidy expenditures [21].

	subsidy expenditures on country food		weight of country food subsidised	
	$	%	kg	%
2011–2012	559	0.001	407	0.002
2012–2013	21292	0.035	9048	0.035
2013–2014	10564	0.017	6659	0.027
2014–2015	39945	0.062	14324	0.056

### Weight of food shipped

According to a 2013 program audit, the annual total weight of food shipped is used by INAC as a measure of the program’s success [12]. The program’s Performance Measurement Strategy uses the volume of subsidised foods, along with the percent of the population reporting excellent or very good health, as the means to assess the program’s progress toward its “ultimate outcome: isolated northern communities’ nutritional choices and community health are strengthened” [15].[p. 12] One criterion for judging whether NNC is achieving this objective is whether annual per capita shipments of subsidised foods are “stable or increasing from the 2011 baseline year” [15].[p. 22]

**Figure 3.**
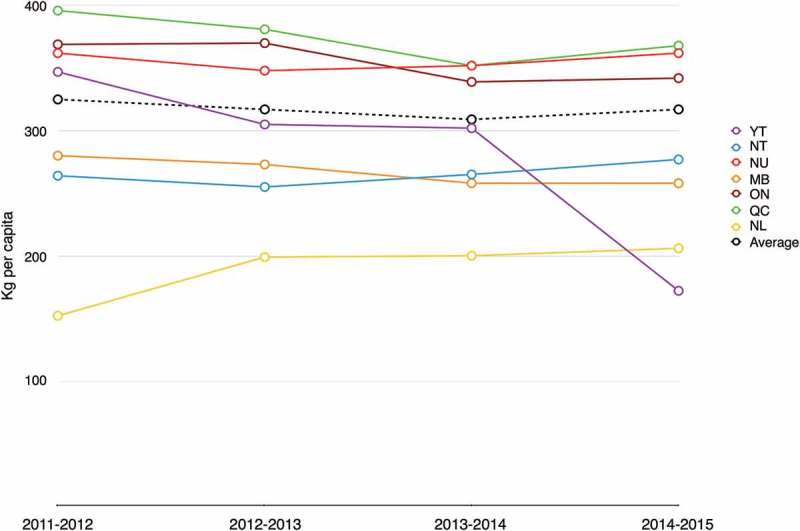
Annual per capita subsidy shipments (kg)* for communities eligible for full subsidy** by province/territory. *Calculated from per capita shipments (kg) and 2011 population estimates23 **Data omitted for Quebec North Shore communities, which receive subsidy for limited periods annually; no shipments reported for Trout Lake NT over the period 2011–2015

**Figure 4.**
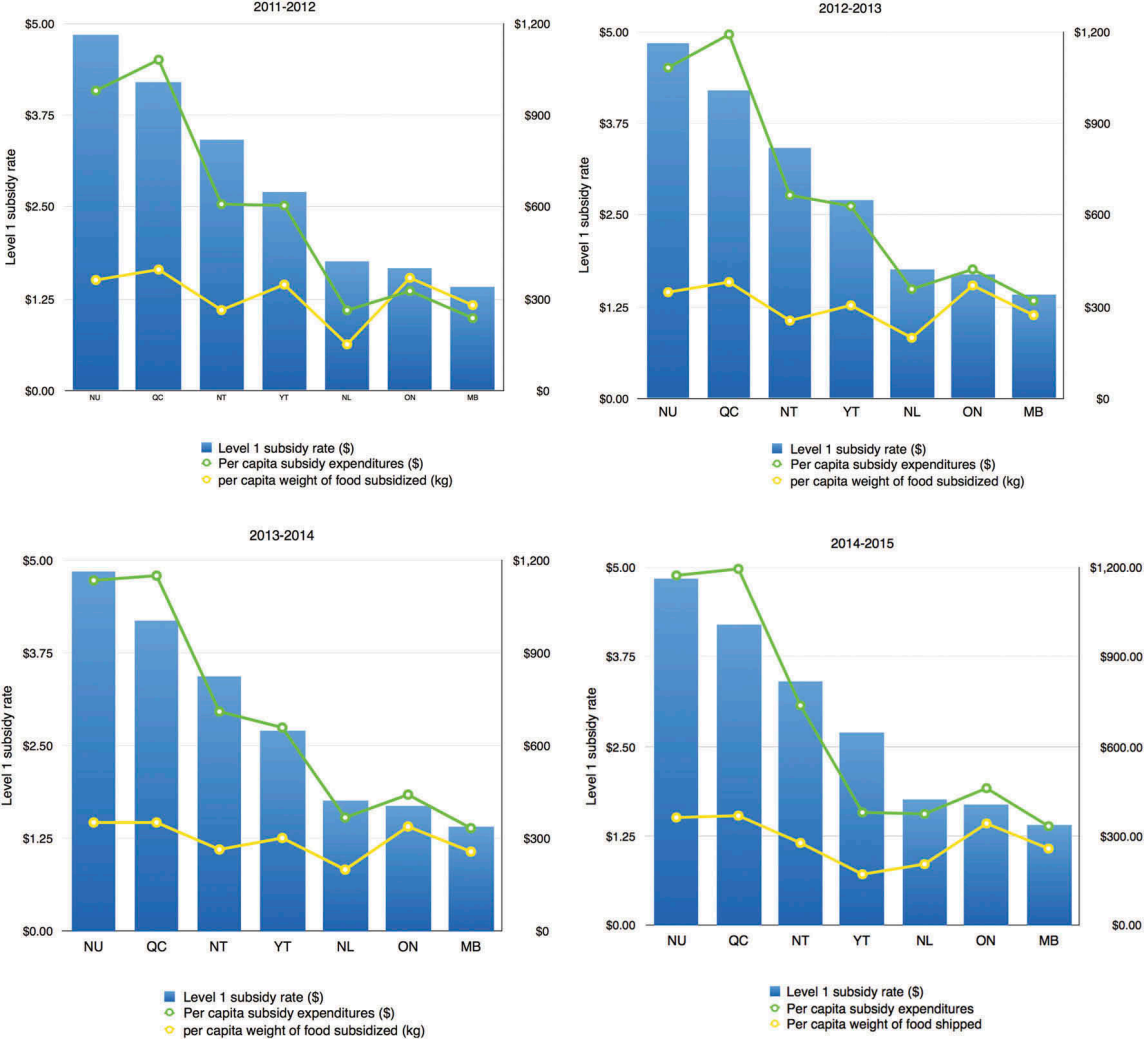
Annual per capita subsidy expenditures ($), weight of food shipped (kg) [21], and Level 1 subsidy rates [17] by province/territory 2011–2015.

Annual fiscal reports provide the total and per capita weight of subsidised food shipped to communities [21]. Although the overall average weight of subsidised shipments is relatively stable over the period 2011–2015, there is regional variability, both in the weight of annual per capita shipments and the trend over time (Figure 3). Communities in Nunavut, Ontario and Quebec all have annual per capita shipments that exceed the overall average; in contrast, communities in Northwest Territories, Manitoba and Newfoundland receive lower-than-average per capita shipments. Annual per capita shipments to Old Crow, Yukon have fallen from 347 kg in 2011–12 to 172 kg in 2014–15 [21]. Figure 4 compares per capita subsidy expenditures and weights with average subsidy rates in provinces and territories. In Nunavut, Quebec, Northwest Territories, Yukon, and Newfoundland & Labrador, there is a clear gradient across measures, with higher per capita subsidy expenditures and weights recorded in communities with higher subsidy rates. Ontario and Manitoba are exceptions to this pattern.

### Verification of reporting accuracy

According to an internal audit of NNC conducted in June 2013, the program outsources claims processing through a competitive process to a third-party company [12]. Retailers send claims and supporting documentation (invoices and waybills labeled by category of eligible items, destination community and store) to claims processors who are instructed by INAC to perform one review per month for each retailer, on a random sample of 50 lines of supporting documentation. The 2013 audit found that INAC had not exercised the “right to audit” clause contained in its contract with the claims processor, nor had it requested information on the processor’s quality assurance strategy. The 2014 audit of NNC by the Office of the Auditor General of Canada did not include auditing of claims processing in its mandate [3].

### Food cost estimates

In its 2016 Performance Measurement Strategy, INAC employs price trends and averages in the cost of the Revised Northern Food Basket (RNFB) as the means of monitoring program success [15]. The RNFB is a food costing tool developed by Health Canada in 2007 [25]. It replaces an earlier version, the Northern Food Basket, which critics claimed lacked emphasis on lean meats, fresh fruit and vegetables. The RNFB is designed to reflect the purchasing patterns of a family of four individuals (1 man and 1 woman aged 25–49 years, 1 boy aged 13–15 years, and 1 girl aged 7–9 years) for one week, and is concordant with the most recent dietary recommendations of the US Dietary Reference Intakes [26] and *Canada’s Food Guide for First Nations, Inuit and Métis* [24].

RNFB cost estimates are compiled from price information on a pre-determined list of 67 food items with 5% of the total cost added to the estimate for miscellaneous items [25]. In the case of NNC, RNFB cost estimates are calculated by retailers: “Northern retailers must…be able to provide electronically, as per a predetermined schedule and format, monthly food pricing reports by eligible community for a pre-determined list of items (i.e. Revised Northern Food Basket)” [18]. According to program documents, the accuracy of reported prices can be verified by program officials during compliance reports and audits, and retailers must “agree to provide access to facilities and records for recipient audit purposes” [18] at the request of program officials.

Quarterly RNFB cost data are available for fiscal years 2011–12 through 2014–15 [27–30]. The 2011–12 RNFB cost report lists the retailer(s) that supplied the information for each community; it also contains the following statement: “Price data from Arctic Co-operatives Ltd. for co-ops in Nunavut and the Northwest Territories (NT), Rampart Rentals for Norman Wells, NT, and Stanton Group for Aklavik and Tuktoyaktuk, NT, was either not available for all periods, or could not be used to accurately calculate the cost of the RNFB and/or draw comparisons” [27]. Subsequent RNFB cost reports do not list the retailers that supplied the estimates.

There are 35 communities which report subsidy expenditures during fiscal years 2011–12 through 2014–15 for which RNFB cost estimates are not available [27–30]. Nine of these are Quebec communities along the North Shore of the St. Lawrence River, which are eligible for subsidy during the Spring months only when ice conditions prevent marine access. These Quebec North Shore communities account for a relatively low proportion of NNC program expenditures: $349,602 over the period 2011–2015 or 0.144% of total subsidy funds [21]. No RNFB estimates are available for an additional 11 full-subsidy and 15 partial-subsidy communities for which the program reports regular subsidy expenditures.^3^ ^3^No subsidy expenditures are reported for Trout Lake and Gametì NT, Black Lake and Stony Rapids SK, and Lourdes-de-Blanc-Sabon QC 2011–2015 though these communities are eligible for partial subsidy [21].


The 11 full-subsidy communities that lack RNFB cost estimates are: Sachs Harbour and Colville Lake NT; Grise Fiord, Resolute, Kugaaruk and Whale Cove NU; Big Trout Lake and Muskrat Dam ON; and Natuashish, Postville and Black Tickle NL).^4^ ^4^RNFB cost estimates for Old Crow YT are unavailable for March 2015 [30]. Retailers in these communities received combined subsidies averaging $3.5 million annually or a total of $14.8 million over the period 2011–15 [21]. There are no RNFB cost estimates for 15 partial-subsidy communities in NT, SK, MB, ON and QC. In total, retailers in all subsidised communities that lack RNFB estimates receive subsidies averaging $3.8 million annually or a total of $15.5 million over the period 2011–2015.

**Figure 5.**
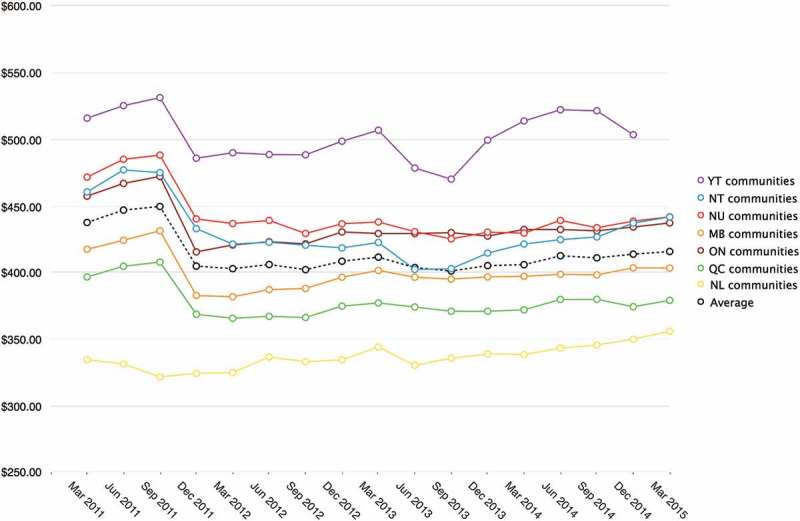
Average RNFB costs* 2011–2015 for communities eligible for full subsidy** by province/territory [27–30]. *RNFB costs are supplied by retailers.20 **Cost estimates are unavailable for 10 of the 69 eligible communities. Partial data are available for several of the remaining communities: No data are available for Pauingassi MB for June-December, 2011; No data are available for Rigolet NL for March 2011; No data are available for Old Crow, Yukon for March 2015. Data for Quebec North Shore communities are not included as they are served by NNC for short periods only when there is no marine service available.

**Figure 6.**
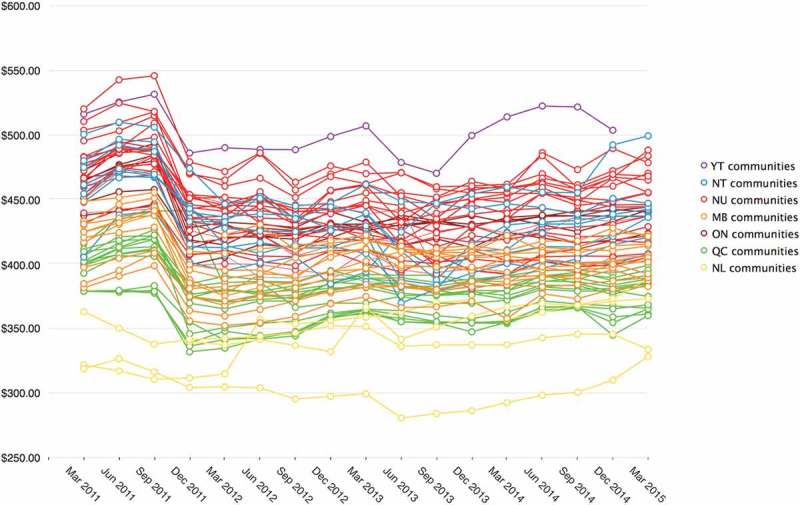
Average RNFB cost* 2011–2015 for communities eligible for full subsidy** by community [27–30]. * RNFB costs are supplied by retailers.20 **Cost estimates are unavailable for 10 of the 69 eligible communities. Partial data are available for several of the remaining communities: No data are available for Pauingassi MB for June-December, 2011; No data are available for Rigolet NL for March 2011; No data are available for Old Crow, Yukon for March 2015. Data for Quebec North Shore communities are not included as they are served by NNC for short periods only when there is no marine service available.

Comparison of quarterly RNFB costs reveals an initial rise over the period March through September 2011 followed by a drop in all regions in March 2012 (Figure 5). Comparison of RNFB costs across communities indicates this pattern is no aberration (Figure 6). Since March 2012, RNFB costs have remained relatively stable in most regions, with the exception of Yukon, where costs have varied considerably. Compared to the overall average, RNFB costs in eligible communities are consistently higher in Yukon, Northwest Territories, Nunavut and Ontario than they are in Manitoba, Quebec, and Newfoundland & Labrador.

### Retailer compliance reviews

According to the program website, retailers who receive payment under the NNC subsidy are subject to periodic third-party compliance reviews. These reviews are available on the program website [21]. The original intent of retailer compliance monitoring was to conduct a biennial audit of each retailer who received more than 1% of the subsidy. This intent was reflected in the first 2 years of implementation: there were seven compliance audits conducted in 2011–12 and seven more conducted in 2012–13. Since that time, there have only been four additional compliance reports conducted, in 2013–14. The current description of NNC’s compliance monitoring process is as follows:

Each year, a sample of Northern retailers and Southern suppliers are chosen to undergo a compliance review. This process can help determine whether they are complying with the terms and conditions of the funding agreement they signed with Indigenous and Northern Affairs Canada…and are transferring the subsidy to customers [16].

The published compliance reviews have found retailers in violation of the terms of their agreements on multiple occasions. For example, in 2012 a retailer was found to have claimed level 1 subsidy rate for an item for a four month period, when that item was only eligible for the lower level 2 subsidy rate; the retailer was billed for the difference [31]. In 2013 a retailer was found to have been using 2 distinct sets of weights to calculate the costs charged to consumers and the amount of subsidy claimed; as well, the retailer failed to provide waybills along with freight invoices [32].

### Recent developments

The transition from Food Mail to NNC was in part an effort at cost containment. NNC was implemented with a fixed budget of $60 million annually [11]. Program operation and public education costs aside, the actual funds allocated to subsidy were $53.9 million annually [33].

In 2013 an internal program evaluation acknowledged that cost containment measures within the program ensured this annual limit on expenditures was not exceeded:

…safeguards are in place to mitigate any risk of over spending the $53.9 million. However, as most key informants have pointed out, the capped budget limits any growth in demand resulting from additional communities eligible for the program. The more communities eligible for the subsidy, the fewer subsidy dollars are available. Note that the capped budget also does not account for population growth and increased consumption [33].[p. 45]

INAC officials determined that “the capped budget may not be sufficient to support access to nutritious food” [33].[p. vii] In November 2014, the federal government announced an additional $11.3 million dollars to support NNC subsidies over a two-year period, as well as an additional 5% per year in program funds [34]. The combined increase resulted in total available funds of $65.2 million in 2014–15 and $68.5 million in 2015–16. In its 2016 budget, the federal government committed to provide NNC with ongoing additional funding in the amount of $64.5 million over 5 years ($12.9 million annually) and $13.8 million per year beginning in 2021 [35].

## Discussion

### Program lacks adequate evidence base

The goal of NNC, to provide northerners with affordable access to nutritious perishable food, cannot be adequately assessed using the reporting tools available to the program. The current structure and regulatory framework of NNC do not ensure that program officials, or the public, have access to the information on food availability and cost, by community and by store, that is needed to evaluate whether the program is meeting its goal. Initial increases in weight of food shipped to communities during the implementation period 2011–12 occurred against a backdrop of no subsidy, therefore it is not even possible to compare the efficacy of NNC with that of he previous Food Mail program. Similarly, the only significant food cost reductions over the period 2011–2015 were achieved with the implementation in March 2012, following the unsubsidised period. Subsequent comparisons of later RNFB costs with 2011 costs simply replicate the initial “success” of the program’s implementation.

### Lack of program responsiveness

There are significant disparities between the intentions expressed in program literature and the implementation of NNC. Many of these centre around the NNC program’s lack of responsiveness. Between the April 2011 program launch and June 2016, calls to review community eligibility, subsidy rates, and the list of eligible items have been made by parliamentary review committee [11], the NNC Advisory Board [1], the Nunavut Food Security Coalition [13], the Auditor General of Canada [3], and the UN Special Rapporteur on the Right to Food [2]. Despite these calls, only one change was made to the list of eligible communities (the addition of Pauingassi MB to the list of full-subsidy communities, effective August 2012) and no significant amendments were made to the program until the changes to the list of eligible communities were announced in July 2016 [22]. Subsidy rates have not been altered since the program’s launch, nor has the list of eligible foods, which underwent only slight modification in response to community pressure in November 2011 with the addition of processed cheese spread and bacon. Calls to subsidise household goods, infant care products and personal hygiene items, whose costs pose significant burdens for many northerners, have gone unheeded. Other items formerly subsidised under food mail, such as hunting and fishing equipment and craft items, are not supported under NNC.^5^ ^5^A recent report estimates the cost of an all-season hunting outfit at upwards of $55,000 [36].


Although NNC subsidises retail sales of country food produced and shipped from federally-regulated food processing facilities [23], country food accounts for less than 0.1% of subsidy expenditures annually, making this component of NNC of limited efficacy in promoting availability of traditional foods in northern communities. Calls for the government to reduce regulatory barriers on the transport of harvested foods between provinces and territories and subsidise the cost of purchasing and maintaining harvesting equipment have to date gone unanswered. No explanation has been provided either in parliament or in the media for this period of inertia surrounding the core components of NNC.

### Inequities in the availability of food

Between 2011 and 2015, NNC delivered an average of 317 kilograms per year of subsidised foods to each resident living in eligible communities. But that average obscures considerable variability. Average per capita weight of subsidised foods is consistently higher in Nunavut, Manitoba, Ontario and Quebec than it is in Northwest Territories and Newfoundland & Labrador. Per capita weight of subsidised foods has declined precipitously in Old Crow, Yukon following changes to the community’s retail options.^6^ ^6^In Fall 2014, North West Company store closed its doors [37]. For a period of several months, the community had no retail store. Perishable goods were supplied by a local non-profit agency, or by private shipments. In May 2015, a new Arctic Co-operatives Limited store opened in the community [38]. It is unclear why such regional variability exists among full-subsidy communities. Isolated communities in Yukon, Northwest Territories, Manitoba and Newfoundland & Labrador are no less dependent on the subsidy than those in other regions. Lower per capita food shipments may indicate that NNC is not serving these communities in these regions as well as those in Nunavut, Ontario and Quebec. Recent changes to the list of eligible communities may result in similarly inequitable results if the pattern of regional differences results from factors such as lack of retail competition or prohibitive freight costs.

Community eligibility, high subsidy rates, and consistent per capita tonnage are not adequate to ensure access to fresh commodities in remote communities. A 2015 report by the Nunavut Food Security Coalition expressed grave concerns over the failure of the existing subsidy to ensure the availability of perishable food in some communities [13]. In the High Arctic communities of Resolute Bay, Arctic Bay and Grise Fiord, the combination of limited retail options and weather-related conditions results in limited supply of perishable foods. In winter, weather-related flight cancelations mean stores await re-supply, often for 2–3 week periods. From April to October, temperature fluctuations mean that frozen perishables often thaw en route, necessitating their disposal from risk of spoilage. Direct order and country food purchases are no solution to these challenges as they too must be shipped by air. In 2013, the Grise Fiord co-operative store purchased a refrigerated container to protect the integrity of frozen foods during marine transport. However foods transported via sealift are ineligible for NNC subsidy.

### Inequities in the affordability of food

A major criticism of the old Food Mail subsidy was the lack of equitable pricing that existed across northern communities:

…in general,… as a result of the transportation subsidy, prices for the most nutritious perishable foods are reduced by an average of 15–20% of their non-subsidised prices, although this impact varies widely by community depending on degree of isolation and the types of food offered by retailers [39].

NNC’s available food cost data demonstrates persistent inequities in food pricing between regions and communities. RNFB costs in Canada’s territories are consistently higher than those in other subsidised jurisdictions. The subsidy is expressly designed to iron out the inequities posed by the high logistical and transportation costs of shipping perishable foods to isolated communities. The highest subsidy rates are provided for those communities who bear the highest burden of these logistical costs. The effect of the NNC subsidy should be to produce relatively equitable food pricing across the different regions. The fact that it does not is an indication that it is not working.

Take for example, the RNFB costs reported for the communities of Igloolik and Hall Beach. These two communities in the Qikiqtaaluk Region of Nunavut are located within 70 km of each other. Igloolik has a population of 1454; Hall Beach has a population of 546 (Census of Canada 2011). Both receive similar air service; each has 2 retail stores. Level 1 and 2 subsidy rates for the communities are identical [17]. In the fiscal year 2014–15, per capita subsidy expenditures and per capita kilogram shipments in the communities were comparable [40]. Nevertheless, in every quarter March 2011 through March 2015, the RNFB costs reported by retailers and made available on the NNC website have been 10% higher in Igloolik than those reported in Hall Beach [27–30]. The reason for this discrepancy is inscrutable unless one considers the potential for retailers to price subsidised foods on the basis of factors other than just subsidy applied to wholesale and freight costs. There is no provision within the subsidy to prevent such an occurrence. Consider a 2014 report by the Nunavut Bureau of Statistics which lists the median gross income of residents of Igloolik between 10% and 15% higher than that of residents of Hall Beach over the period 2011–2013 [41]. The observed pattern of food cost inequities is evidence that the NNC program is not serving northern communities in a way that is equitable or just, nor is it preventing retailers from potentially benefiting from subsidy in communities where this is advantageous or profitable.

NNC’s Performance Measurement Strategy contains no criteria for evaluating the prices retailers charge for subsidised foods [15]. There is no mechanism for comparing the prices of individual food items among retailers in the same community or region, nor is there a means of reporting this information to the public. One can see this information would be highly useful to consumers who purchased subsidised goods, and would serve as an effective means of fostering competition and holding retailers accountable for the funds they receive. Prices of subsidised foods are not set to southern benchmarks nor are there ceilings or limits applied and enforced on food pricing.

### Fiscal reporting and program accountability

A major impediment to program accountability is the structure of fiscal reporting. Subsidy claims processing has been outsourced and limited oversight exists over the quality and accuracy of claims processing [12]. While the program reports subsidy allocations by both community and retailer, these tallies are not cross-referenced, so there is no way to ascertain exactly what amounts were received in communities by particular retailers. For example, expenditures are reported by product category, so it is possible to know how many kilograms of fruit and vegetables (and their total dollar value in retail sales) were subsidised by the program, but these figures are not available by community or region. As noted above, the RNFB cost reports provide cost estimates for the total 67-item food basket, not for particular items, so it is difficult to know whether categories of expenditures, or prices for individual items, differ by region and community. After the first annual RNFB report for 2011–12, the retailer providing the information on pricing is not listed, so no comparison can be made by the public among retailers’ pricing patterns.

What is evident from NNC fiscal reports is that a single retailer, North West Company receives the majority of the subsidy. In the fiscal year 2014–15, North West Company received 50% of subsidy funds or $32.8 million [40]. Its nearest competitors, Arctic Co-operatives Limited and Fédération des cooperatives du nord du Québec, received 19% ($12.3 million) and 12% ($7.8 million) respectively. North West Company company operates 139 stores in northern communities [42]. In 2015 North West Company’s Canadian operations generated over $1 billion in revenues and $98 million in earnings before taxes [43]. In March 2016 the company posted a $15 million profit in its 4th quarter alone [44]. In reports to shareholders, company officials describe its Canadian operations as its most profitable sector: “the engine of our Company’s continued growth is our northern Canadian market” [45][p. 4].

### Lack of retail competition in small communities

According to program documents, NNC is based on a “market-driven model which promotes efficiency, cost-effectiveness and transparency” [16]. The subsidy model recognises that private-sector retailers have made significant investments in capital infrastructure, distribution networks, training and human development in the north, and it partners with retailers to leverage these investments in order to offer health food at affordable prices in remote, northern communities. NNC operates on the premise that if we subsidise retailers’ freight costs, retailers will be able to offer healthy food at affordable prices; northern consumers will then choose to shop at the store in their community that offers the best quality food at the lowest prices.

A market-driven retail subsidy requires two things in order to be effective: a competitive marketplace, where consumers can select from among retail options, and a strong regulatory and accountability framework, to mandate and enforce evidence-based targets for system performance. In the case of NNC, it is questionable whether the retail environment in remote communities can be considered in any sense “competitive”. In Nunavut, for example, there are 25 communities reliant on air freight shipments for all perishable commodities except harvested food. Iqaluit, the largest, currently has 5 retail grocery and convenience stores, 4 of them operated by Northwest Company and 1 by Arctic Co-operatives Limited. Outside of Iqaluit, the average number of stores in Nunavut communities is two. Kugaruuk, Whale Cove and Grise Fiord each have one store. The community of Old Crow, Yukon, has one grocery store.

During consultations regarding the transition from Food Mail to NNC, concerns were raised over how a retail subsidy would operate in small communities with only one or two stores [11]. Community members expressed concern that prices would remain higher in communities with limited retail options. Mr. Darius Elias, Member of the Yukon Legislative Assembly, requested that Old Crow be considered a “special case” and granted approval to continue to be funded through a transportation subsidy arrangement, rather than the new retail subsidy [1,11]. A parliamentary review board also recommended Old Crow be considered a special case: “it remains unclear, however, to what extent [eligibility for full subsidy under NNC] would lead to improvements in quality and price for food items offered at the retailer in Old Crow, Yukon” [11][p. 21]. Limited provision was made for Old Crow under NNC: the community has an expanded list of eligible foods that includes a range of canned and dried goods, spices and baking supplies, diapers, laundry soap, and personal hygeine products, all level 2 subsidy items [23]. Nevertheless, the present analysis reveals that the community of Old Crow, with a single retailer, consistently exhibits the highest RNFB cost in Canada, fully 22–27% higher than the average cost of the RNFB in NNC-subsidised communities [27–30].

Data on the number of retailers operating in communities is difficult to locate, and NNC does not report RNFB costs in many of single-retailer communities. Among the three single-retailer communities in Nunavut, for example, there are no RNFB cost estimates available for any of the years NNC has operated, despite the fact that NNC expenditures are reported annually in these communities [21]. Lack of publicly available data makes it extremely difficult to evaluate whether the lack of retail competition makes food costs higher in these communities.

A parliamentary committee reviewing the NNC proposal concluded that preservation of personal orders under NNC would “serve to somewhat mitigate these concerns, such that it would offer a form of competition with the single retailer” [11].[p. 21] The program website continues to reinforce the belief by program officials that the direct order option “helps preserve competition among Northern retailers and provides consumers with flexibility related to special dietary needs” [16]. However, since 2011, direct orders have accounted for less than 3% of total subsidy dollars. The proportion of subsidy allocated to direct orders is not reported by community, so it is impossible to determine whether this option benefits consumers in single-retailer communities. Their low overall contribution to subsidy expenditures means that direct orders do not serve the intended purpose of preserving competition in limited markets. Further, the presence of a North West Company-owned southern supplier on the list of INAC-approved vendors means this option may not be a viable means of preserving competition in the northern retail sector.

### Current structure of food cost reporting fails to ensure accountability

The key measure of NNC’s accountability is the RNFB cost. But limitations in the structure of food cost reporting hamper the ability of program officials to monitor program efficacy and retailer compliance. One considerable barrier to overall program effectiveness appears to be retailer compliance with the food cost reporting. Failure to report RNFB costs for 35 communities represents a significant gap in the program’s accountability. Without RNFB cost estimates, it is difficult to know whether retailers in subsidised communities are using subsidy payments to lower food prices. For example, in 2014–15 fully 1% ($726,438) of total subsidy expenditures was allocated annually to a single retailer operating in remote Grise Fiord NU, the full-subsidy community with the highest level 1 and level 2 subsidy rates [40]. Average annual subsidy expenditures in Grise Fiord were $726,438 in 2014–15, or a total of $2.68 million over the period 2011–2015 [21]. Resolute NU, with the second-highest level 1 and 2 subsidy rates, receives 2% of the total subsidy annually, with annual expenditures of $1.16 million in 2014–15 or $3.89 million over the period 2011–2015. Without available RNFB estimates, there is simply no public accountability for the prices set by retailers in these communities that receive NNC subsidy.

The RNFB tool itself represents an extremely limited instrument for ensuring the accountability of both the program and participating retailers. Prices of individual items within the RNFB are not reported, nor are prices of foods that are not included in the RNFB. Lack of community- and retailer-level data on food costs means researchers and consumers cannot compare prices between communities and stores, and there are no means within the NNC accountability framework to ensure both RNFB and non-food basket items are priced reasonably and equitably across communities.

### Emphasis on program visibility vs. accountability

Statements from an INAC program review indicate that as early as 2009 the federal government was considering shifting from a transportation subsidy to a retail subsidy model:

The review of the Food Mail Program outlined some weaknesses in the program’s design that could be rectified through a formal agreement between INAC and participating retailers. For example, the review identified a lack of awareness among consumers regarding the federal government’s efforts to reduce the cost of nutritious food in isolated northern communities. An agreement with retailers could hold them accountable for:

- Passing on to consumers the subsidy paid by INAC on eligible products by entering a legally binding agreement to a maximum percentage mark-up over the cost-landed price;
- Transporting goods in a covered vehicle to the retail location to improve the quality of perishable food on arrival;
- Displaying Food Mail signs on eligible products to inform consumers of their savings;
- Providing sales data to INAC to better inform future decisions on eligible products; and,
- Continuing to allow INAC officials to undertake food price surveys at retail locations.

Such an agreement could stipulate that in the event of retailer non-compliance, sanctions could be levied. The sanctions could call for a penalty payable to the band, hamlet or municipal office. If INAC chose to implement such an agreement with retailers, it could be challenged and would require consultation with retailers and INAC’s legal advisors to develop terms and conditions. Such a measure would bring additional transparency to the Program [39].[p. 23]

In actuality, the transition to a retail subsidy model was achieved without many of these recommendations brought to fruition. There is no maximum percentage mark-up for subsidised foods; in the majority of communities, covered transport is not available. In contrast, early concerns with the subsidy program’s reputation in northern communities have resulted in concerted and coordinated effort on the part of both INAC and retailers to improve both the visibility and reputability of the subsidy.

In 2008, the Devolution and Territorial Relations Branch at INAC established an interdepartmental research team to examine available research on the Food Mail program [39]. In August of that year, INAC appointed a Ministerial Special Representative, Mr. Graeme Dargo,^7^ ^7^The introduction to Dargo’s 2008 Food Mail Program Review states that “the author has no vested interest in any organization, airline or retail chain associated with this review” [19].p. [3] A 2001 media report entitled “Last of the Bay Boys” describes Graeme Dargo as a former 10-year employee of the North West Company [46]. to engage with communities, senior government and industry officials “to seek views and perspective on the program and potential alternatives” [39].p[19] Four months later, in his report, the representative expressed frustration with what he perceived as a lack of awareness about the Food Mail subsidy in communities:

During my community visits it became apparent that Canada receives no credit for its investment and that community residents are unaware of the Program… Due to the lack of public information and marketing the Government of Canada receives no credit for sizeable [sic] investment of public funds in the Program. Not one of the groups I met with had an inkling of the substantial investment that Canada currently provides to support the Food Mail initiative [19].[p. 15]

In 2011, a Report of the Standing Committee on Aboriginal Affairs and Northern Development stated that one of the main issues related to the operation of the Food Mail program was “awareness: no formal mechanism exists to ensure awareness of the program and its impacts on food availability and affordability” [11].[p. 11]

Visibility is a requirement built into the NNC program. In order to be eligible to receive subsidy payments, northern retailers must: “agree to make the program visible and the subsidy transparent to consumers through messages on cash register receipts and communication material, in-store signage and displays provided by [INAC]” [18]. In response to community members’ concerns over whether the subsidy was really being passed on to consumers, in 2012 the NNC Advisory Board recommended retailers make the program “more visible in-store” [1].[p. 12] The results of these recommendations are visible in the program’s logic model, published in January 2016 [15], and in retail stores in the form of NNC signs that display the prices of items before and after subsidy is applied. Beginning April 2016, this before/after subsidy information is also provided on grocery receipts [47].

However neither the signs nor the receipts provide any new or substantive information. Lists of eligible communities and foods along with community-specific level 1 and 2 subsidy rates have been available on the NNC program website since the program was implemented in 2012. The signs and receipts simply express the per kg subsidy rate for that community as it is applied to a particular grocery item. While that information is arguably more accessible to northern consumers now than it used to be on the program website, it represents no increase in the transparency of NNC, nor an increase in retailers’ accountability for passing on the value of the subsidies they receive in the form of lower food prices. To be clear, both the in-store signs and point-of-sale receipts are measures that increase the *visibility* of NNC but do not address the program’s fundamental lack of *accountability*.

What is missing is a full and substantive accounting of the true wholesale and freight costs borne by retailers, and the means by which these costs are reflected in food pricing. During parliamentary review of the NNC proposal, INAC staff stated that contribution agreements with participating retailers would contain the necessary oversight and leverage to enforce retailer accountability [11]. However in April 2013, the UN Special Rapporteur on the Right to Food, Olivier de Schutter, submitted a report to the UN General Assembly in which he observed: “Nutrition North Canada currently publishes the subsidy level per kilogram for each eligible community, but it does not require retailers to inform [INAC] or the public of their airfreight costs. As such, the federal Government has no way of verifying if the subsidy is being passed on” [2].[pp. 17–18] According to the Auditor General of Canada:

Overall, we found that Aboriginal Affairs and Northern Development Canada has not verified whether the northern retailers pass on the full subsidy to consumers. The Department has not required the information it needs to verify this in the contribution agreements it has signed with northern retailers. It also has not required that compliance reviews of northern retailers include analysis of profit margins in order to verify that the full subsidy is being passed on. This finding is important because passing on the full subsidy to consumers is a program requirement, and is necessary to make nutritious food more accessible and affordable to Northerners [3].[Chapter 5, p. 5]

It is unlikely such information will be made available, and there is no regulatory mechanism for enforcing those disclosures from private-sector retailers. Without such a measure of accountability within the NNC program, northern retailers exercise total and arbitrary power over food pricing. This was illustrated by a February 2016 announcement by North West Company that it was reducing prices on fresh produce in nine Nunavut communities [48]. No information was given on how those communities were selected, what process informed the selection of individual food items, or how price reductions were determined. North West Company spokesperson Derek Reimer “wouldn’t say whether the lower prices are here to stay, but he says the company will keep an eye on the situation” [48]. When asked by a CBC reporter whether the competitor would follow suit, Arctic Co-op spokesperson Duane Wilson stated “naturally we expect to see that member co-ops would follow suit” [48]. Under NNC, decisions about food pricing – pricing subsidised foods in subsidy-eligible communities – are entirely within the control of retailers, and there is currently no mechanism within the NNC program to influence or mandate price controls.

Finally, the system of retailer compliance reporting implemented in 2011 has not been carried out. Within the current structure of NNC, retailer compliance reporting represents a significant check on retailer activities. Though the information collected is partial and supplied by the retailers themselves, compliance audits nevertheless represent one of the only means the program has of evaluating retailer operations with respect to the subsidy. If compliance reports aren’t conducted, this means is ineffective. No compliance reports have been published on the program website since March 2014 [21]. According to the program website, there has been no compliance report for the largest recipient of the subsidy, North West Company, since December 2012 [21]. Failure to comply with the program’s original schedule of compliance reporting, whether caused by lack of oversight or resources, significantly impacts program performance and accountability.

In its methodology, the present study was limited to use of publicly-available materials. This was not for lack of attempting a larger, more comprehensive investigation of the implementation of NNC involving interviews with consumers, retailers and program staff, community and store visits. Despite widespread support among Indigenous leadership and community groups, funding applications for this work were unsuccessful. Use of fiscal and program reports limits the scope of this inquiry into what is publicly available, however this is an advantage for a federal program that values transparency and accountability. Gaps in available data, such as missing retailer identifiers in RNFB cost reports and inconsistent scheduling of compliance reports, highlight areas of focus for program officials seeking to make NNC responsive to northern residents. Independent collection of food price data, in subsidised and unsubsidised remote northern communities and on a wide range of staple food and non-food items, would assist evaluators in determining whether NNC and other food security measures are reducing the financial burden of high grocery prices on northern residents and their families.

## Conclusion

This analysis examines NNC’s intended outcome – improved access to perishable, nutritious food – in terms of the quality of service delivered to northern populations, with emphasis on population coverage and equity. The existing retail subsidy does not ensure northerners have access to nutritious, healthy food in a manner that is fair and equitable across regions and communities. Food prices are high, and there is a consistent pattern of price inequities (higher in the territories) that is not alleviated by current subsidy rates. These problems are compounded by the failure of the program to respond to the concerns about community eligibility, subsidy rates, eligible foods, and retailer accountability that have been raised by community members, critics, the Auditor General of Canada, and the program’s own Advisory Board.

Most concerning is the lack of accountability within the NNC program for northern retailers. Subsidy claims are processed using limited amounts of randomly-selected data to verify the accuracy of claims. Food cost estimates are unavailable for many communities in which retailers receive subsidies, including some single-retailer communities where consumers are highly vulnerable to retail price fluctuations. Lack of detailed fiscal reporting makes it difficult for consumers to compare food availability and affordability by community and by store. A limited number of retailer compliance audits have been undertaken, none recently.

All of this occurs against a backdrop of extremely high food insecurity in remote, northern communities. This food insecurity is compounded by recent evidence that climate change is reducing the availability of traditionally harvested country foods [49], thus increasing northerners’ dependency on retail stores as a reliable source of affordable food in communities. The current structure of the NNC program – a market-driven retail subsidy – assumes a competitive marketplace that does not exist in the majority of northern Canadian communities. Northern consumers represent a captive and highly vulnerable market, one that the subsidy should be designed to serve. In the absence of fundamental changes to the program’s structure and operation, increases to the program’s overall budget are unlikely to result in improved food availability or food cost equity.

The question remains whether improved service can be achieved through program modification alone or whether more substantive restructuring is necessary. Given the limited competition in most northern communities, it is certain that the current subsidy requires both a more rigorous system of retailer accountability and a strong regulatory framework for food pricing. The challenge is to determine whether these mechanisms can be incorporated into a subsidy model whose driving ethos is based on a market competition. It may be necessary to consider alternative policy models such as those operating in Alaska and Greenland.

## Supplementary Material

Supplementary Table 1: Eligible communities, subsidy levels (full or partial) and subsidy rates (Levels 1 and 2)

## Declaration

The views expressed in the following manuscript are solely those of the author. The author has no competing interests and was not contracted or paid by any organization to conduct this evaluation.

## References

[CIT0001] Government of Canada (2012). First report of the advisory board, Nutrition North Canada, for the period February 2011 to March 2012, Cat. No. R71-74/2012E-PDF.

[CIT0002] De Schutter O. (2016). Office of the United Nations High Commissioner for Human Rights, Mandate of the Special Rapporteur on the right to food: preliminary remarks on the visit to Canada from 6 to 16 May 2012. http://www.srfood.org/en/official-reports.

[CIT0003] Office of the Auditor-General of Canada (2014). Report of the Auditor-General of Canada. Chapter 6: nutrition North Canada - Aboriginal Affairs and Northern Development Canada. http://www.oag-bvg.gc.ca/internet/docs/parl_oag_201411_06_e.pdf.

[CIT0004] Statistics Canada (2013). Cansim Table 105-0546: household food insecurity. http://www.statcan.gc.ca/pub/82-625-x/2013001/article/11889-eng.htm.

[CIT0005] Wallace S (2012). Inuit health: selected findings from the 2012 Aboriginal Peoples Survey. Statistics Canada, Cat. No. 89-653-X-No. 003.

[CIT0006] Alaska Department of Health and Social Services (2015). Food stamp benefits. http://dhss.alaska.gov/dpa/Pages/fstamps/default.aspx.

[CIT0007] Kalaallit Niuerfiat (2016). About Pilersuisoq. http://www.kni.gl/en/vores-virksomheder/om-pilersuisoq/.

[CIT0008] Indigenous and Northern Affairs Canada (2016). News release: nutrition North Canada wants to hear from Northerners. Press release.

[CIT0009] National Collaborating Centre for Indigenous Health Policy (2013). Briefing note: public policy models and their usefulness in public health: the stages model. Report for the Institut national de santé publique, Québec. http://www.ncchpp.ca/docs/ModeleEtapesPolPubliques_EN.pdf.

[CIT0010] Hardee K, Laili I, Ron M Linking health policy with health systems and health outcomes: a conceptual framework.

[CIT0011] House of Commons Canada (2016). from food mail to nutrition North Canada: report of the standing committee on aboriginal affairs and Northern Development, March 2011. http://www.parl.gc.ca/HousePublications/Publication.aspx?DocId=4823376.

[CIT0012] Aboriginal Affairs and Northern Development Canada (2013). Internal audit report: audit of Nutrition North Canada. Prepared by Audit and Assurance Branch, Project No. 13-48. https://www.aadnc-aandc.gc.ca/eng/1375887194752/1375887248731.

[CIT0013] Nunavut Food Security Coalition (2015). The Nutrition North Canada Program. Report prepared by the Niqittiavak Committee December 2013.

[CIT0014] Government of Nunavut (2016). Economic data: 2014 nunavut food price survey detailed data tables. Compiled by the Nunavut Bureau of Statistics, Department of Executive and Intergovernmental Affairs; 2014. http://www.gov.nu.ca/eia/information/economic-data.

[CIT0015] Indigenous and Northern Affairs Canada (2016). Performance Measurement Strategy 4.1.2 Nutrition North Canada, CIDM #7924093. https://www.aadnc-aandc.gc.ca/eng/1416929989252/1416930028365.

[CIT0016] Indigenous and Northern Affairs Canada (2016). How Nutrition North Canada works. http://www.nutritionnorthcanada.gc.ca/eng/1415538638170/1415538670874.

[CIT0017] Indigenous and Northern Affairs Canada (2016). Nutrition North Canada: eligible communities. http://www.nutritionnorthcanada.gc.ca/eng/1415540731169/1415540791407.

[CIT0018] Indigenous and Northern Affairs Canada (2016). Nutrition North Canada: information for retailers and suppliers. http://www.nutritionnorthcanada.gc.ca/eng/1415626422397/1415626591979.

[CIT0019] Dargo G (2008). Food mail program review: findings and recommendations of the minister’s special representative. Dargo & Associates. http://caid.ca/FoodMailRev123108.pdf.

[CIT0020] Indigenous and Northern Affairs Canada (2016). Nutrition North Canada: registered Southern suppliers that take direct or personal orders. http://www.nutritionnorthcanada.gc.ca/eng/1415814013968/1415814031766.

[CIT0021] Indigenous and Northern Affairs Canada (2016). Nutrition North Canada: reports. http://www.nutritionnorthcanada.gc.ca/eng/1415647255632/1415647437113.

[CIT0022] Indigenous and Northern Affairs Canada (2016). News release: government of Canada expands Nutrition North Canada program to isolated communities in the North. 18 July 2016, Inuvik NT. http://news.gc.ca/web/article-en.do?nid=1100309&tp=1.

[CIT0023] Indigenous and Northern Affairs Canada (2016). Nutrition North Canada: eligible food. http://www.nutritionnorthcanada.gc.ca/eng/1415548276694/1415548329309.

[CIT0024] Health Canada Eating Well with Canada’s Food Guide.

[CIT0025] Indian Affairs and Northern Development Canada (2007). The Revised Northern Food Basket. Catalogue No. R3-56/2007E-PDF. http://publications.gc.ca/site/eng/317335/publication.html.

[CIT0026] Otten JJ, Hellwig JP, Meyers LD (2006). Dietary reference intakes: the essential guide to nutrient requirements.

[CIT0027] Indigenous and Northern Affairs Canada (2015). Cost of Revised Northern Food Basket in 2011-2012. http://www.nutritionnorthcanada.gc.ca/eng/1369313901161/1369313917620.

[CIT0028] Indigenous and Northern Affairs Canada (2015). Cost of Revised Northern Food Basket in 2012-2013. http://www.nutritionnorthcanada.gc.ca/eng/1369313792863/1369313809684.

[CIT0029] Indigenous and Northern Affairs Canada (2015). Cost of Revised Northern Food Basket in 2013-2014. http://www.nutritionnorthcanada.gc.ca/eng/1429275989528/1429276029787.

[CIT0030] Indigenous and Northern Affairs Canada (2016). Cost of Revised Northern Food Basket in 2014-2015. http://www.nutritionnorthcanada.gc.ca/eng/1458130696862/1458130716818.

[CIT0031] Samson & Associates (2016). Compliance review report - North West Company LP, December 7, 2012. http://www.nutritionnorthcanada.gc.ca/eng/1411152419722/1411152435672.

[CIT0032] Samson & Associates (2016). Compliance review report - Arctic Co-operatives Limited, December 2013. http://www.nutritionnorthcanada.gc.ca/eng/1411144279766/1411144351602.

[CIT0033] Aboriginal Affairs and Northern Development Canada (2016). Implementation evaluation of the nutrition North Canada Program. Prepared by evaluation, performance measurement and review branch, audit and evaluation sector project No. 1570-7/12023, September 2013. https://www.aadnc-aandc.gc.ca/eng/1395347953550/1395348287432.

[CIT0034] Government of Canada (2016). Budget 2016. http://www.budget.gc.ca/2016/docs/plan/toc-tdm-en.html.

[CIT0035] Indigenous and Northern Affairs Canada (2016). News release: government of Canada expands Nutrition North Canada program to isolated communities in the North, Inuvik NT, 18 July 2016. http://news.gc.ca/web/article-en.do?nid=1100309&tp=1.

[CIT0036] Campbell M, Honrado L, Kingston B (2014). Action Canada Report: hunger in Nunavut: local Food for Healthier Communities. http://www.actioncanada.ca/project/hunger-nunavut-local-food-healthier-communities/.

[CIT0037] Dolphin M (2016). Old Crow residents left without a grocery store, Yukon News, 21 November 2014. http://yukon-news.com/news/old-crow-residents-left-without-a-grocery-store/.

[CIT0038] CBC News Old Crow, Yukon’s most northern town, welcomes Co-op store, 19 May 2015. http://www.cbc.ca/news/canada/north/old-crow-yukon-s-most-northern-town-welcomes-co-op-store-1.3078357.

[CIT0039] Indian and Northern Affairs Canada (2016). Food mail review: interim report. Prepared by devolution and territorial relations branch, March 2009. Cat. No. R3-99/1-2009E. http://caid.ca/FoodMailIntRev031509.pdf.

[CIT0040] Government of Nunavut (2016). Economic Data: Nunavut median total income of tax filers with income by region and community 1999-2013. Compiled by the Nunavut Bureau of Statistics, Department of Executive and Intergovernmental Affairs; 2014. http://www.gov.nu.ca/eia/information/economic-data.

[CIT0041] Indigenous and Northern Affairs Canada (2016). Reports: 2014-2015 Full Fiscal Year. http://www.nutritionnorthcanada.gc.ca/eng/1453219591740/14532197656759.

[CIT0042] North West Company (2016). Operations: Canada retail banners. http://www.northwest.ca/operations/canada.php.

[CIT0043] North West Company (2016). Top work, top results: the North West Company Inc. Annual Report 2015, 08 April 2016. http://www.northwest.ca/content/annual_filings/2015_YE_-_Annual_Report_-_APR8-16.pdf.

[CIT0044] North West Company (2016). News release: the North West Company Inc. announces fourth quarter earnings, a quarterly dividend and the refinancing terms of its Canadian and International loan facilities, Winnipeg MB, 15 March 2016. http://www.northwest.ca/content/news_releases/Q1_2016_Press_Release_-_JUN8-16.pdf.

[CIT0045] North West Company (2016). A time to invest: the North West Company Inc. Annual Report 2014, 09 April 2015. http://www.northwest.ca/content/annual_filings/2014_YE_-_Annual_Report_-_APR9-15.pdf.

[CIT0046] Gleeson R (2016). Last of the Bay Boys: northern adventure continues for Scottish immigrant. Northern News Services, Yellowknife NT, 29 Oct 2001. http://www.nnsl.com/frames/newspapers/2001-10/oct29_01dar.html.

[CIT0047] Aboriginal Affairs and Northern Development Canada (2016). News release: harper Government implements new measure to increase transparency of Nutrition North Canada for consumers through amended funding agreements, Ottawa ON, 01 April 2015. http://news.gc.ca/web/article-en.do?nid=957869&tp=1.

[CIT0048] CBC News (2016). North west Company drops produce prices in 9 communities, 04 February 2016. http://www.cbc.ca/news/canada/north/nwc-price-drops-in-nunavut-1.3433367.

[CIT0049] Rosol R, Powell-Hellyer S, Chan HM (2016). Impacts of decline harvest of country food on nutrient intake among Inuit in Arctic Canada: impact of climate change and possible adaptation plan. Int J Circumpolar Health.

[CIT0050] Government of Canada (1985). Department of Indian Affairs and Northern Development Act, R.S.C.. http://laws-lois.justice.gc.ca/eng/acts/i-6/.

