# Supplementary material for: Canada’s northern food subsidy Nutrition North Canada: a comprehensive program evaluation
Source: Int J Circumpolar Health. 2017 Feb 2;76(1):1279451. doi: 10.1080/22423982.2017.1279451 (PMC5328347; doi:10.1080/22423982.2017.1279451)
Supplement: Supplementary Table 1: Eligible communities, subsidy levels (full or partial) and subsidy rates (Levels 1 and 2) [file zich_a_1279451_sm7099.docx]

**Supplementary Table 1: Eligible communities, subsidy levels (full or partial) and subsidy rates (Levels 1 and 2)^18^**

| Subsidy level | Province/Territory | Community | Level 1 subsidy | Level 2 subsidy |
| --- | --- | --- | --- | --- |
| Full | YT | Old Crow | $2.70 | $0.90 |
|  | NT | Aklavik | $1.60 | $0.05 |
|  |  | Paulatuk | $3.80 | $2.00 |
|  |  | Sachs Harbour | $6.10 | $4.30 |
|  |  | Tuktoyaktuk | $2.50 | $0.70 |
|  |  | Ulukhaktok (Holman) | $4.50 | $2.70 |
|  |  | Trout Lake | $3.20 | $1.40 |
|  |  | Colville Lake | $5.20 | $3.40 |
|  |  | Deline | $2.70 | $0.90 |
|  |  | Fort Good Hope | $2.90 | $1.10 |
|  |  | Norman Wells | $2.20 | $0.40 |
|  |  | Tulita | $2.70 | $0.90 |
|  | NU | Arctic Bay | $8.60 | $6.80 |
|  |  | Cape Dorset | $4.60 | $2.80 |
|  |  | Clyde River | $6.60 | $4.80 |
|  |  | Grise Fiord | $16.00 | $14.20 |
|  |  | Hall Beach | $5.60 | $3.80 |
|  |  | Igloolik | $5.60 | $3.80 |
|  |  | Iqaluit | $2.30 | $0.50 |
|  |  | Kimmirut | $5.40 | $3.60 |
|  |  | Pangnirtung | $4.10 | $2.30 |
|  |  | Pond Inlet | $8.10 | $6.30 |
|  |  | Qikiqtarjuaq | $4.80 | $3.00 |
|  |  | Resolute | $10.20 | $8.40 |
|  |  | Sanikiluaq | $1.80 | $0.05 |
|  |  | Cambridge Bay | $1.80 | $0.05 |
|  |  | Gjoa Haven | $3.40 | $1.60 |
|  |  | Kugaaruk | $4.10 | $2.30 |
|  |  | Kugluktuk | $2.60 | $0.80 |
|  |  | Taloyoak | $3.70 | $1.90 |
|  |  | Arviat | $2.00 | $0.20 |
|  |  | Baker Lake | $3.30 | $1.50 |
|  |  | Chesterfield Inlet | $3.20 | $1.40 |
|  |  | Coral Harbour | $4.10 | $2.30 |
|  |  | Rankin Inlet | $2.20 | $0.40 |
|  |  | Repulse Bay | $4.20 | $2.40 |
|  |  | Whale Cove | $2.80 | $1.00 |
|  | MB | Gods Lake Narrows | $1.20 | $0.05 |
|  |  | Gods River | $1.20 | $0.05 |
|  |  | Island Lake (Garden Hill) | $1.60 | $0.05 |
|  |  | Lac Brochet | $1.40 | $0.05 |
|  |  | Little Grand Rapids | $1.20 | $0.05 |
|  |  | Negginan (Poplar River) | $1.30 | $0.05 |
|  |  | Oxford House | $1.40 | $0.05 |
|  |  | Red Sucker Lake | $1.60 | $0.05 |
|  |  | St. Theresa Point | $1.60 | $0.05 |
|  |  | Waasagomach | $1.60 | $0.05 |
|  |  | Pauingassi^**^ | $1.20 | $0.05 |
|  | ON | Attawapiskat | $1.40 | $0.05 |
|  |  | Bearskin Lake | $1.30 | $0.05 |
|  |  | Big Trout Lake | $1.60 | $0.05 |
|  |  | Fort Albany | $1.30 | $0.05 |
|  |  | Fort Severn | $2.60 | $0.80 |
|  |  | Kashechewan | $1.30 | $0.05 |
|  |  | Muskrat Dam | $1.50 | $0.05 |
|  |  | Peawanuck | $2.40 | $0.60 |
|  | QC | Akulivik | $4.60 | $2.80 |
|  |  | Aupaluk | $4.60 | $2.80 |
|  |  | Inukjuak | $2.90 | $1.10 |
|  |  | Ivujivik | $5.50 | $3.70 |
|  |  | Kangiqsualujjuaq | $4.30 | $2.50 |
|  |  | Kangiqsujuaq | $5.20 | $3.40 |
|  |  | Kangirsuk | $5.40 | $3.60 |
|  |  | Kuujjuaq | $2.60 | $0.80 |
|  |  | Kuujjuarapik | $2.20 | $0.40 |
|  |  | Puvirnituq | $3.90 | $2.10 |
|  |  | Quaqtaq | $5.50 | $3.70 |
|  |  | Salluit | $5.20 | $3.40 |
|  |  | Tasiujaq | $4.30 | $2.50 |
|  |  | Umiujaq | $2.40 | $0.60 |
|  |  | Chevery^***^ | $2.30 | $0.50 |
|  |  | Gethsémani (La Romaine)^***^ | $2.30 | $0.50 |
|  |  | Harrington Harbour^***^ | $2.30 | $0.50 |
|  |  | La Tabatière^***^ | $2.30 | $0.50 |
|  |  | Mutton Bay^***^ | $2.30 | $0.50 |
|  |  | Saint-Augustin-Saguenay^***^ | $2.30 | $0.50 |
|  |  | Tête-à-la-Baleine^***^ | $2.30 | $0.50 |
|  | NL | Hopedale | $1.70 | $0.05 |
|  |  | Makkovik | $1.50 | $0.05 |
|  |  | Nain | $2.50 | $0.70 |
|  |  | Natuashish | $2.00 | $0.20 |
|  |  | Postville | $1.60 | $0.05 |
|  |  | Rigolet | $1.30 | $0.05 |
|  |  | Black Tickle | $1.70 | $0.05 |
| Partial | NT | Gametì (Rae Lakes) | $0.05 | $0.05 |
|  |  | Lutsel k'e | $0.05 | $0.05 |
|  |  | Wha Ti | $0.05 | $0.05 |
|  | SK | Black Lake | $0.05 | $0.05 |
|  |  | Fond-du-Lac | $0.05 | $0.05 |
|  |  | Stony Rapids | $0.05 | $0.05 |
|  | MB | Berens River | $0.05 | $0.05 |
|  |  | Bloodvein | $0.05 | $0.05 |
|  |  | Shamattawa | $0.05 | $0.05 |
|  | ON | Angling Lake | $0.05 | $0.05 |
|  |  | Kasabonika | $0.05 | $0.05 |
|  |  | Kingfisher Lake | $0.05 | $0.05 |
|  |  | Pikangikum | $0.05 | $0.05 |
|  |  | Sachigo Lake | $0.05 | $0.05 |
|  |  | Weagamow Lake | $0.05 | $0.05 |
|  |  | Wunnummin Lake | $0.05 | $0.05 |
|  | QC | Blanc Sablon^**^ | $0.05 | $0.05 |
|  |  | Kegaska^**^ | $0.05 | $0.05 |
|  |  | Lourdes-de-Blanc-Sablon^**^ | $0.05 | $0.05 |

^*^Pauingassi became eligible for full subsidy in August 2012; prior to that, it was eligible for partial subsidy under NNC.

^**^These Quebec communities along the North Shore of the St Lawrence River and Gulf of St Lawrence are eligible for subsidy only during months when there is no marine ferry service. Eligibility is determined on an annual basis depending on ice conditions.

^***^On July 18, 2016, the Government of Canada announced that the list of eligible communities would be expanded;^19^ subsidy levels for these newly-qualified communities have not yet been published.
